# Impact behaviour of 3D printed cellular structures for mouthguard applications

**DOI:** 10.1038/s41598-022-08018-1

**Published:** 2022-03-07

**Authors:** John Saunders, Maria Lißner, David Townsend, Nik Petrinic, Jeroen Bergmann

**Affiliations:** Department of Engineering Science, University of Oxford, Oxford, OX1 3PJ UK

**Keywords:** Engineering, Materials science

## Abstract

Ethylene-Vinyl Acetate (EVA) is the most popular material for manufacturing mouthguards. However, EVA mouthguards are problematic, for example inconsistent thicknesses across the mouthguard. Additive manufacturing provides a promising solution to this problem, as it can manufacture mouthguards with a greater precision. This paper compares the energy dissipation of EVA, the current material used for mouthguards, to various designs of a 3D printed material, some of which contain air cells. Impact testing was carried out at three different strain rates. The Split-Hopkinson bar was used for medium and high strain rate tests, and an Instron test rig was used for low strain rate testing. The best performing design dissipated 25% more energy than EVA in the medium and high strain rate testing respectively while the low strain rate testing was inconclusive. This research has shown that additive manufacturing provides a viable method of manufacturing mouthguards. This opens up the opportunity for embedding electronics/sensors into additive manufactured mouthguards.

## Introduction

Physical exercise is known to have wide ranging benefits to health, for example, decreasing the risk of illness such as cardiovascular disease, type 2 diabetes, specific types of cancer, and osteoporosis^1^. A number of sports and physical activities put the participant at risk of sustaining concussions and orofacial injuries^2^. This can occur in contact sports such as rugby and boxing, or through sports where a hard ball could impact the orofacial region, such as hockey or lacrosse. However, orofacial injuries are also prevalent in non contact sports, such as cycling^3^, occurring due to large impact falls. Orofacial injuries include soft tissue lacerations, damaged teeth, and mandibular/maxillary fractures. It is clear therefore, that measures should be put in place in order to reduce the risk of inujry, particularly as a traumatic dental injury may require expensive treatment and can lead to long-term appearance^4^.

A mouthguard is the most widely used piece of equipment to mitigate the risk of dental injury during sport activities. A mouthguard is an oral device which fits, most commonly, over the maxillary arch and reduces the transmitted force on impact. Bimaxillary mouthguards are also available. However, these are bulky and have large effects on the user’s ability to breathe and speak. These negative effects clearly need to be minimised in order to facilitate wider adoption of this equipment by athletes. Mouthguards are not only worn in sport; other applications include treatment of bruxism and sleep apnoea^5–7^.

The three main types of mouthguard are: pre-fabricated, mouth-formed (also known as boil and bite) and custom-made. Pre-fabricated mouthguards are preformed and sold ready to wear. This means that they are not specifically fitted to the user’s teeth and soft tissue. They are sold as a range of sizes and the user chooses the size which gives the best fit. Mouth-formed mouthguards are made from a thermoplastic material. The user heats up the mouthguard by placing it in boiling water, making the material mouldable. The mouldable mouthguard is then put over the teeth, with pressure applied from biting force, such that it forms to the shape of the user’s teeth and soft tissue. Custom-made mouthguards are formed in a dental laboratory using an impression of the user’s teeth, taken by a dentist. They are usually made using a thermoplastic called polyethylene vinyl acetate (EVA). A model of the user’s teeth is created using the impression. A sheet of EVA is then heated up and formed over this model. Pre-fabricated mouthguards are cheap. The fit of the mouthguard and the protections it offers however, are poor^8^. It is accepted that custom-made mouthguards offer the highest level of protection^9,10^ and the best fit. However, their main drawbacks include the expense^8^ and, in most cases, the need for at least one dental appointment. Home-kits do exist for custom-made mouthguards. This does require familiarity with the process of obtaining an impression.

The first known instance of mouthguard use in sport was in boxing, where boxers would create their own from materials such as cotton, tape, sponge or wood. However, as the boxer would have to clench his jaw to keep these in place, these solutions detracted from performance^2^. The first appropriate development of the mouthguard was in the 1890s when Woolfe Krause placed strips of gutta percha, a thermoplastic latex that resembles rubber, over the boxer’s teeth^11^. This practice developed over the following years until a fight between Ted Lewis and Jack Britton led to the officials deeming that a mouthguard violated the rules of the game. It was not until 1927 that the New York State Athletic Commission allowed boxers to wear mouthguards again. This was due to the extent of injuries sustained during a fight between Jack Sharkey and Mike McTigue^2^

Today, mouthguards are commonplace in many sports. In the UK, their use is mandatory in ice hockey, fencing, boxing, lacrosse^12^ and is recommended for rugby^13^. Such sports that require mandatory use of mouthguards have shown a reduction in dental injuries of 60%^12^.

Moreover, the use of wearable technology is already becoming commonplace in sport, with some professional rugby players wearing GPS and heart monitoring systems during matches^14^. Players from Saracens, a professional English rugby team, are now wearing sensor patches behind their ears to measure the force experienced during collisions^14^. As a mouthguard is already worn by many players in sport, it is a good option for recording information such as head acceleration^15^ and breathing rate^16^. Accelerometers are able to provide real-time data to estimate player exposure. They do not yet however, have the required sensitivity to directly measure concussions^17^. Therefore, performance tracking is likely to be more robust than concussion monitoring. The technology required for this must be incorporated into the mouthguard safely and as unobtrusively as possible^18^. Additive manufacturing is a potential option for achieving this, as it will allow the cavity for the smart technology to be printed precisely, minimising the excess material.

Westerman et al.^19^ carried out a study to see whether or not structured air inclusions in a sheet of EVA would increase energy dissipation. The study impact tested 4 different EVA sheets; a solid EVA sheet as the control and 3 EVA sheets with varying air cell size and wall thickness between air cells. The study found that the EVA sheet with the largest air cells (3x3x2mm) and smallest wall thickness (1mm) produced the largest reduction in force (32%) compared to the EVA sheet with no air inclusions. This suggests that if it is possible to put air inclusions into a mouthguard material, it will potentially increase the amount of energy it can dissipate. This will become more important as smart technology in mouthguards becomes more prevalent, which leads to cavities around the integrated technology. Nonetheless, creating and/or monitoring air cavities in EVA sheets under controlled conditions is challenging.

Another problem reported in literature with thermoforming EVA is uneven thinning of the material. Del Rossi et al.^20^ reported that the thickness of the EVA reduced, on average, by 46% on the occlusal surface overlying the molars and between 47% and 60% along the labial surface of the central incisors and canines. This is due to sagging of the EVA sheet at its centre when it is heated up. The sagging of the material causes it to thin. This thin material then forms the part of the mouthguard on the occlusal surface^20^. Takeda et al.^21^ reported that insufficient occlusal protection in a mouthguard, such as thinning of the material, is likely to cause mandibular fracture when the user is hit in the jaw. To counteract this thinning, EVA mouthguards are made with thicker sheets to provide sufficient protection in all areas. This means however, there is excess material in other areas, causing issues.

Additive manufacturing of mouthguards can provide a solution to these problems. Using additive manufacturing to produce the mouthguard, it is possible to precisely control the shape and thickness of the mouthguard at any point. This will allow the entire mouthguard to be of optimal thickness; areas where protection is needed the most will have a larger thickness, whereas other areas can be thinner, increasing comfort and breathability, as well as having an optimal thickness around any electrical components to create a more suitable smart mouthguard. Additive manufacturing will also allow for the precise addition of air cells in the formed mouthguard, which could increase the energy dissipation of the mouthguard. Nevertheless, a fundamental understanding of the mechanical performance of additive manufactured devices is required. This is of high importance if the components have to be resistant to impact scenarios and at the same time be able to accommodate sensor systems.

Consequently, this research aims to investigate the strain rate dependent mechanical performance of different additively manufactured design specimens and their influence on the dissipated energy. Firstly, experiments are conducted with solid design specimens for two different materials—a 3D printable thermoplastic elastomer and the EVA material traditionally used for mouthguards. Secondly, the suitability of the 3D printed material compared to the traditional material is evaluated in strain rate-dependent loading environments. Thirdly, the influence of different air cell structures (solid, cubic and spherical) on the dissipated energy is investigated. Finally, experimental observations of the influence of material and specimen designs on the dissipated energy as a function of strain rate are discussed.

## Methods

### Materials and specimen design

#### Material selection

The most commonly examined physical properties of a mouthguard are energy dissipation, hardness, stiffness, tear strength, tensile strength and water absorption. This paper will focus on energy dissipation, as this is the fundamental property relating to the protective capability of a mouthguard.

For a new material to be viable for the use of mouthguards, its mechanical behaviour must be compared against the current best material used since no standards for mouthguards materials are available. This means when choosing a material to 3D print mouthguards, it is important to choose a material with a similar Young’s modulus to EVA, the current material used in mouthguards. In literature, however, the Young’s modulus of the EVA is rarely reported. Therefore, hardness was used as the quantity to compare the new material to EVA. Tensile strength was not considered in this case because the thickness of the EVA sheets varied between studies. This means that the values are not comparable as Tran et al.^22^ showed that the tensile strength reduces significantly as the thickness increases.

The mean Shore A hardness of EVA, using values measured in seven different studies is 82^22–27^. Arnitel ID 2045 Natural (DSM, The Netherlands), is a thermoplastic copolymer TPE and has a Shore D hardness of 34. According to ASTM D2240, this is equivalent to a Shore A hardness of 83, and so is similar to EVA examined in the seven studies. Arnitel is also biocompatible and non-toxic and so will be safe to use as an oral device.

#### Specimen design

A study by Westerman et al.^28^ found that the optimal thickness for an EVA mouthguard is 4mm. If the mouthguard is thinner than 4mm, it will dissipate significantly less energy. If the mouthguard is thicker than 4mm, the increase in energy dissipation is insignificant and user comfort would be decreased. For this reason, it was decided that the thickness of the compression samples would be 4mm with a diameter of 12mm.

EVA and Arnitel specimens with no air inclusions (solid) were tested with the aforementioned dimensions. The EVA samples were taken from a 4mm thick EVA sheet using a 12mm diameter punch, struck with a hammer. The EVA samples were not thermoformed.

Furthermore, 3D printed specimens with air inclusions are considered to understand their mechanical behaviour with respect to dissipated energy. This fundamental understanding is crucial when considering smart devices such as mouthguards with included sensors for performance tracking. While smart technologies can enhance product safety, the mechanical performance related safety is not allowed to be compromised.

As mentioned before, a study found that air inclusions in EVA sheets had a beneficial effect on energy dissipation^19^. The study compared specimens with cuboid air cells of different sizes and with different wall thickness between adjacent air cells. It found that the sample with the largest air cells and smallest wall thickness had the greatest energy dissipation. The dimension of the air cell across the thickness of the samples was 2mm. This allowed 1mm of solid material between the edge of the air cell and the surface of the specimen.

**Figure 1:**
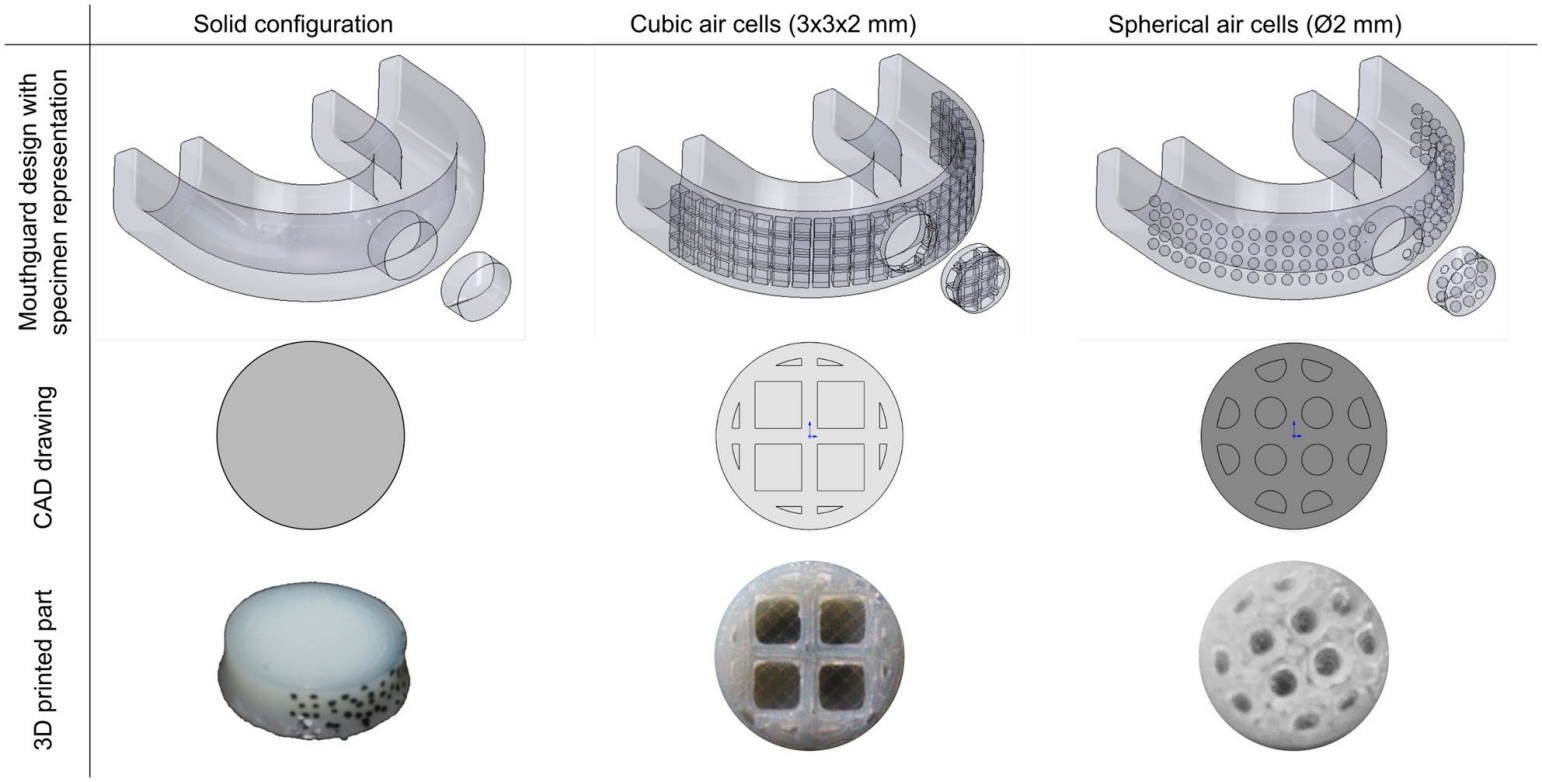
Overview of potential mouthguard designs with specimen representations, cross sections of CAD drawings and 3D printed specimens.

It was therefore decided that the dimensions of the air inclusions in one of the samples would be that of the best performing sample in Westerman et al.’s study^19^. This is a cuboid air cell where the height and width are 3mm while the depth is 2mm with 1mm wall thickness between adjacent air cells. It was noted that 3D printing would allow for a larger variety of shapes of air cells than when using EVA. In order to determine the effectiveness of different shapes of air cell specimens with spherical air cells are also be tested. These specimens would have a 2mm diameter ($\phi$2mm) air cells with 1mm wall thickness. This size was chosen to be consistent with having 1mm wall thickness between the air cell and the surface of the specimen. Figure 1 presents the specimen geometries.

#### Specimen manufacturing

The three different specimen types (solid, cubic and spherical) made out of Arnitel were manufactured using the additive manufacturing method Fused Deposition Modelling (FDM). FDM is a low cost and high speed method to 3D print^29,30^. As printing mouthguards is something that needs to be viable on a commercial scale, these features will be of interest. This method also has the ability to produce products which have a high strength with good precision^31^. FDM also has little to no waste material, if no support structure is required, as only the required material is fed through the nozzle. This is a key benefit of FDM, compared to other methods which require a reservoir of material.

The two printers available are the Ultimaker 3x and the Creality CR10. Early prints were made using the Ultimaker 3x, which uses a Bowden extruder. Using this machine however, the print quality was poor, with unwanted gaps in between the air cells. This occurred because of a lag due to the length of the tube between the motor and the nozzle.

The final products were printed using the Creality CR10, which has a direct drive extruder, with settings as follows: nozzle diameter 40mm, extrusion multiplier 90%, layer thickness 0.1 mm, infill 100%, overlap between outline and infill 5%, nozzle temperature 230 $^{\circ }$C, bed temperature 60 $^{\circ }$C, cooling fan 20%, overall print speed 20 mm/s. It should be noted that coasting was disabled as this caused larger gaps in the print. The extrusion multiplier and infill were set to optimise the print quality between the air cells. An extrusion multiplier below 90% would lead to larger gaps between air cells, and above 90% would lead to material being printed in the air cells. The effect of increasing or decreasing the print speed was similar. As the motor is directly attached to the nozzle, the filament is pulled into the nozzle, causing it to be in tension. This means that there is no lag between the motor and nozzle and so the print quality is much higher.

When the material is printed onto the print bed, it is in liquid state. For an elastic material, like Arnitel used for this paper, it contracts when it cools. This causes it to curl up at the ends. This is due to the material not adhering to the glass bed. This can be solved by putting professional painters tape on the glass bed. This has a textured surface, helping the material to adhere to the base. The CAD drawings for the specimens with air cell inclusions along with a cross section of the 3D printed specimens can be seen in Fig. 1 alongside with some potential designs for 3D printed mouthguards. While in the real-world the air cells are not visible unless the mouthguard or specimens are cut, the CAD drawings of the potential mouthguard design are shown transparent to provide a better understanding.

The thickness of both the 3D printed and EVA specimens was consistently less than 4mm. Table 1 shows the minimum, maximum, mean and standard deviation of thickness for each specimen configuration. 9 samples were measured for each specimen configuration. The same 9 samples were used for the impact testing. For each specimen, the thickness was measured at 3 distinct points and an average was taken.

**Table 1:** Measurements of specimen thickness for 9 samples of each specimen configuration.

Material	EVA	Arnitel ID 2045 natural		
Internal structure	Solid	Solid	Cubic air cell	Spherical air cell
Minimum thickness (mm)	3.560	3.620	3.610	3.600
Maximum thickness (mm)	3.670	3.820	3.780	3.780
Mean thickness (mm)	3.600	3.710	3.680	3.660
Standard deviation	0.042	0.060	0.058	0.052

### Experimental test setups

Experiments are performed as a function of strain rate to: (1) understand the mechanical performance of two different materials produced using two different manufacturing technologies, (2) investigate the impact of three different cell structures on dissipated energy, and (3) evaluate the suitability of 3D printed materials. Compression tests in through thickness direction of the specimens are performed to obtain representative mechanical information when impact scenarios on the mouthguard are considered—* e.g.* hockey ball impact, head to ground impact. To represent a wide range of strain rates, three different strain rates are defined. In this way, a clear dependence of the different materials and specimen configurations on the strain rate is achieved.

#### High-strain rate testing setup

The Split Hopkinson Pressure bar (SHPB) was used to characterise the energy dissipation of the specimens for high-strain rate experiments. The SHPB is a well established test setup to characterise materials at high-strain rates. Other dynamic test setups such as the Charpy Impact test are believed to provide quantitative results rather than qualitative results due to the existence of dynamic effects such as inertia.

The SHPB works as follows: a gas gun is loaded to a predetermined pressure. This is used to fire the striker at a velocity associated with the pressure in the gas gun. The striker then impacts the input bar generating, in the case of this experiment, a compressive stress wave. This compressive stress wave propagates along the input bar until it reaches the specimen, causing the specimen to be loaded and deform. The stress wave is then partially reflected back to the input bar and partially transmitted to the output bar^32^.

**Figure 2:**
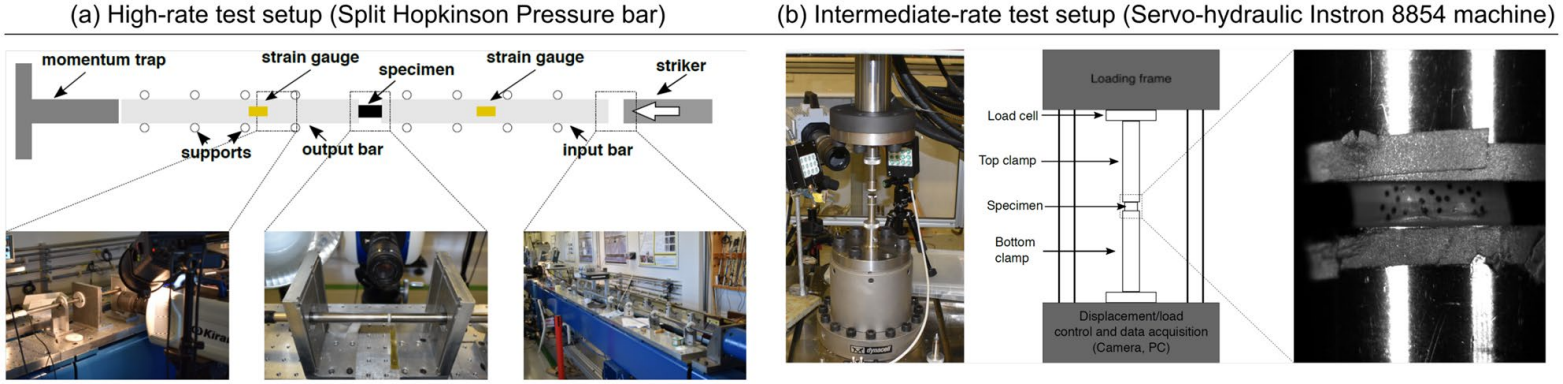
Graphical illustration of (**a**) the Split Hopkinson Pressure bar setup and (**b**) the Instron 8854 testing setup.

A diagram of the set up used can be seen in Fig. 2a. The specimen, illustrated as a black rectangle, can be seen sandwiched between the input and the output bar. Both the input and the output bar are instrumented with strain gauges and are supported on low friction bearings. These bearings prevent movement of the bars in any direction other than axial, whilst allowing free movement in the axial direction. A Wheatstone bridge amplifier is used to convert the variation of resistance from the strain gauges to a voltage signal, which is recorded using an oscilloscope.

The SHPB loads the specimen at very high strain rates, and the stress propagation along the specimen is finite. This means the time taken for the stress wave to propagate along the specimen is significant with respect to the timescale of the deformation. Therefore, initially the specimen is not in dynamic equilibrium as it is only experiencing stress on the side of the input bar, not at the output bar. It is only when the stress along the specimen becomes uniform that the results can be considered valid.

The following assumptions are made in order to analyse the data:

1. The strains measured by the strain gauges on the surface of the bar provide an accurate representation of the strains inside the bar in the case of dynamic equilibrium.
2. The shape of the stress wave remains constant between the strain gauges and the specimen.

The first assumption is easy to satisfy providing the strain gauges are placed far enough away from the end of the bar. The typical distance used for this is at least 10 times the diameter of the bar. The second assumption can be satisfied if a short striker bar is used, meaning wave dispersion can be neglected.

The testing set up is shown in Fig. 2a and the bar data is as follows: both the input and output bar material was titanium with a density of 4430 kg/m$^{3}$ and a diameter of 16 mm, strain gauges 1 and 2 were on the input bar and were 2.110 m and 0.227 m from the specimen respectively, strain gauge 3 was on the output bar and was 0.410 m from the specimen, the striker length was 2.500 m, the input bar length was 2.500 m and the output bar length was 2.500 m.

Testing on the SHPB was done at two different strain rates. For the lower strain rate test, the gas gun was loaded to 0.7 bar. At this pressure, the striker was fired at 5 m/s. For the higher strain rate test, the gas gun was loaded to 1.5 bar. At this pressure, the striker was fired at 10 m/s.

When carrying out each test, the specimen was sandwiched between the input and output bars, with particular care taken to ensure the specimen was in the centre of the bars. A high speed Kirana camera (Specialised Imaging, UK) was used to take photographs of the test, at 200,000 fps and 500,000 fps for the 5 m/s and 10 m/s tests respectively. Before each test, the camera was used to take photographs of the specimen to ensure the correct operation of the diagnostic equipment. Good quality photographs of the test are important as it allows the point at which the specimen is no longer constrained by the bars to easily be seen. The measurements from the strain gauges were recorded on an oscilloscope. Three specimens for each specimen configuration were tested.

#### Low-strain rate testing setup

In order to get full material characterisation at a range of strain rates, low strain rate compression testing was completed on the Instron 8854 (Instron, USA) with a hydraulic testing rig, as shown in Fig. 2b. Two EVA and three Arnitel specimens for each design were tested.

The testing on the Instron rig was carried out at a speed of 40mm/s and the data (*e.g.* force and displacement) was collected using test expert, by Instron. High speed photographs were taken on a Photron camera (Photron, USA) at a frame rate of 10,000 fps.

#### Data acquisition method

The comparison of the strain-rate dependent mechanical response of different cellular structures and material types follow the conventional approach of recording the force-displacement curve from which the nominal stress-strain relationship is derived as

1 $$\begin{aligned} \sigma _{ENG}=\frac{F}{A_{0}} \end{aligned}$$

for the nominal stress and by

2 $$\begin{aligned} \epsilon _{ENG}=\frac{u}{L_{0}} \end{aligned}$$

for the nominal strain, where F is force, A$_{0}$ denotes the initial area, u is the displacement and L$_{0}$ denotes the initial thickness of the sample. The nominal stress-strain relationship is predominantly considered for the determination of the elastic behaviour (Youngs’ modulus).

To investigate the suitability of the 3D printed material in comparison to the EVA material, true stress-strain relationships are believed to be more suitable due to the hyperelastic nature of the rubber-like materials. The true stress is calculated as

3 $$\begin{aligned} \sigma _{TRUE}=\sigma _{ENG}(1-\epsilon _{ENG}) \end{aligned}$$

while the true strain is obtained as follows

4 $$\begin{aligned} \epsilon _{TRUE}=\ln (1-\epsilon _{ENG}) \end{aligned}$$

assuming conservation of volume and uniform deformation. Because of that, test results in which the specimens are within the bar diameter for the high-rate experiments are considered.

While this is straightforward for the low strain rate experiments in which the force-displacement history is a direct output, a comprehensive analysis of the SHPB experiments is required which is based on the stress wave propagation theory^32^. This is explained in detail in the following.

The SHPB can be modelled as a series of thin rods, which deform a small specimen using stress waves. The stresses and particle velocities at the specimen bar interface can be calculated by measuring the stress waves in the input and output bars using the attached strain gauges. These can then be used to calculate the stress and strain rate histories of the specimen^32^.

If precise strain gauges of short gauge lengths are attached to the surface of the bar, the axial elastic stress and particle velocities, *v*, in the cross section of the bar where the strain gauges are attached can be calculated using Eqs. 5 and 6

5 $$\begin{aligned} \sigma (x_{G},t)=\alpha (x_{G},t)+\beta (x_{G},t)=E\epsilon (x_{G,t}) \end{aligned}$$

6 $$\begin{aligned} v(x_{G,t})=\frac{\alpha (x_{G},t)-\beta (x_{G},t)}{\rho c} \end{aligned}$$

In these equations, *E* is the Young’s modulus of the bar material, measured in Pa, $\alpha (x_{G},t)$ and $\beta (x_{G},t)$ are the forward and backwards travelling stress waves respectively as a function of position, *x*, measured in m, and time *t* measured in s, with G denoting the position of the strain gauges. $\rho$ is the density of the bar material, measured in kg/m$^{3}$, and *c* is the elastic stress wave speed for the bar material, measured in m/s, which can be calculated using the following equation

7 $$\begin{aligned} c=\sqrt{\frac{E}{\rho }} \end{aligned}$$

It is possible to calculate the magnitude of the forwards travelling ($\alpha (x,t)$) wave and reflected ($\beta (x,t)$) waves as a function of position and time using the method of characteristics. This enables the calculation of total stress at any point in the bar, including the downstream end of the bar by measuring the strain at the positions of the strain gauges. Equations 8 and 9 can then be used to calculate the specimen extension and the specimen resistance force respectively. INP and OUT refer to the input and output bars, SPEC refers to the cross section of the input or output bar closest to the specimen and A refers to the cross section of the input or output bars.

8 $$\begin{aligned} \Delta u(t)=\int _{0}^{t} \frac{\beta (x^{INP}_{SPEC},t)-\alpha (x^{INP}_{SPEC},t)}{\rho c} d\tau - \int _{0}^{t} \frac{\beta (x^{OUT}_{SPEC},t)-\alpha (x^{OUT}_{SPEC},t)}{\rho c} d\tau \end{aligned}$$

9 $$\begin{aligned} F(t)=\frac{1}{2}\left\{ A_{INP}[\alpha (x^{INP}_{SPEC},t)+\beta (x^{INP}_{SPEC},t)]+A_{OUT}[\alpha (x^{OUT}_{SPEC},t)+\beta (x^{OUT}_{SPEC},t)]\right\} \end{aligned}$$

The stresses and strains within the specimen now need to be calculated. F$_{T}$, F$_{R}$ and F$_{I}$ refer to the transmitted, reflected and incident forces respectively. L$_{0}$ is the initial specimen thickness, and u$_{1}$ and u$_{2}$ are the displacements of the input and output bars respectively.

For this, it is first necessary to calculate the strain rate as a function of the particle forces at the specimen-bar interfaces. $\dot{u}$ denotes the derivative of bar displacement, *u*, with respect to time - the bar velocity.

10 $$\begin{aligned} {\dot{\epsilon }}_{ENG}(t)=\frac{\dot{u}_{1}(t)-\dot{u}_{2}(t)}{L_{0}} \end{aligned}$$

The particle velocity in a travelling wave is related to stress, for $\frac{dx}{dt}=\mp c$, by:

11 $$\begin{aligned} \sigma = \pm \rho c v = \pm \rho c \dot{u}(t) = Z\dot{u}(t) \end{aligned}$$

where Z is the acoustic impedance of the material.

The particle velocities at the specimen-bar interface are given by:

12 $$\begin{aligned} \dot{u}_{1}(t) = - \frac{F_{I}(t-\delta t)}{Z_{B}A_{B}} + \frac{F_{R}(t+\delta t)}{Z_{B}A_{B}} \end{aligned}$$

13 $$\begin{aligned} \dot{u}_{2}(t) = - \frac{F_{T}(t+\delta t)}{Z_{B}A_{B}} \end{aligned}$$

where Z$_{B}$ and A$_{B}$ refer to the acoustic impedance and area of the bars respectively and $\delta t$ is the time shift between the gauges and the specimen. Combining Eqs. 11 to 13 gives Eq. 14.

14 $$\begin{aligned} {\dot{\epsilon }}_{ENG}(t) = \frac{-F_{I}+F_{R}+F_{T}}{Z_{B}A_{B}L_{0}} \end{aligned}$$

When the material is in dynamic equilibrium, the forces on the specimen-bar interface at input and output are equal. Therefore, $F_{T}=F_{I}+F_{R}$ such that the strain rate in the specimen can be given by:

15 $$\begin{aligned} {\dot{\epsilon }}_{ENG}(t)=\frac{2F_{R}}{Z_{B}A_{B}L_{0}} \end{aligned}$$

and finally the strain in the specimen can be calculated using Eq. 16.

16 $$\begin{aligned} \epsilon _{ENG}(t) = \int _{0}^{t} \frac{2F_{R}}{Z_{B}A_{B}L_{0}} d\tau \end{aligned}$$

The stress in the specimen is more simple to calculate. It is found by dividing the transmitted force by the specimen area, as shown in Eq. 17.

17 $$\begin{aligned} \sigma _{ENG}(t)=\frac{F_{T}(t+\delta t)}{A_{spec0}} \end{aligned}$$

The true stress-strain relationship is then obtained using the Eqs. 4 and 3.

#### Data post-processing

When choosing the total test time there are two things to consider. The first is that the data is only valid while the diameter of the specimen does not exceed the diameter of the bars. Secondly, a mouthguard is required to be worn over an entire season and after each impact, it must return to its initial shape. Therefore, it is necessary that results are only considered for elastic behaviour of the material. Both materials are considered ideal for wearable protective equipment due to their highly flexible nature and therefore a high elastic limit is apparent. Nevertheless, this study’s focus is predominantly in the understanding of the suitability of a 3D printed material compared to a conventional material. Therefore, it is believed that the time at which the specimen diameter is still within bar diameter provides a sufficient limit in which the material is within it’s elastic behaviour. However, separate research would be required to confirm this. To allow a direct comparison between the different specimen configuration tests, the shortest valid test time is found and used as the total test time for all tests at that speed. Different test times are used for each test speed.

The final quantity to calculate was the energy dissipated by the specimen. The energy dissipated by a material undergoing displacement is given by Eq. 18. In the script, the force and displacement values are stored in vectors. Therefore numerical integration was required. This was done using the *cumtrapz* function in Matlab which carries out a cumulative numerical trapezoidal integration of two vectors.

18 $$\begin{aligned} W=\int F \cdot du \end{aligned}$$

## Results and discussion

**Figure 3:**
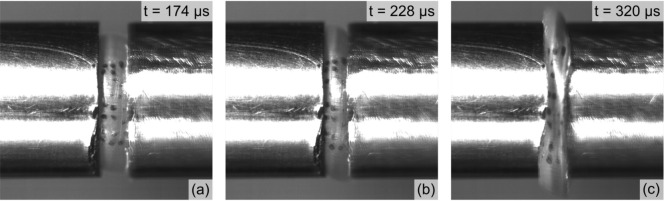
Representative Kirana high speed images of one high strain rate experiment where specimen diameter is; within bar diameter (left), equal to bar diameter (middle) and greater than bar diameter.

For the high strain rate experiments, all specimen diameters exceed the bar diameter after a certain impact time. This can be seen in Fig. 3 which shows points at which the specimen diameter is less than, equal to and greater than the bar diameter for 10 m/s EVA test.

The data processing for the high- and medium-rate experiments is carried out up to an impact time of 360 $\upmu$s for the 5 m/s tests and 150 $\upmu$s for the 10 m/s tests. This corresponds to the specimen deformation well within the bar diameter. The corresponding true strain is at 40%. This value is used as the maximum true strain for all experiments.

**Figure 4:**
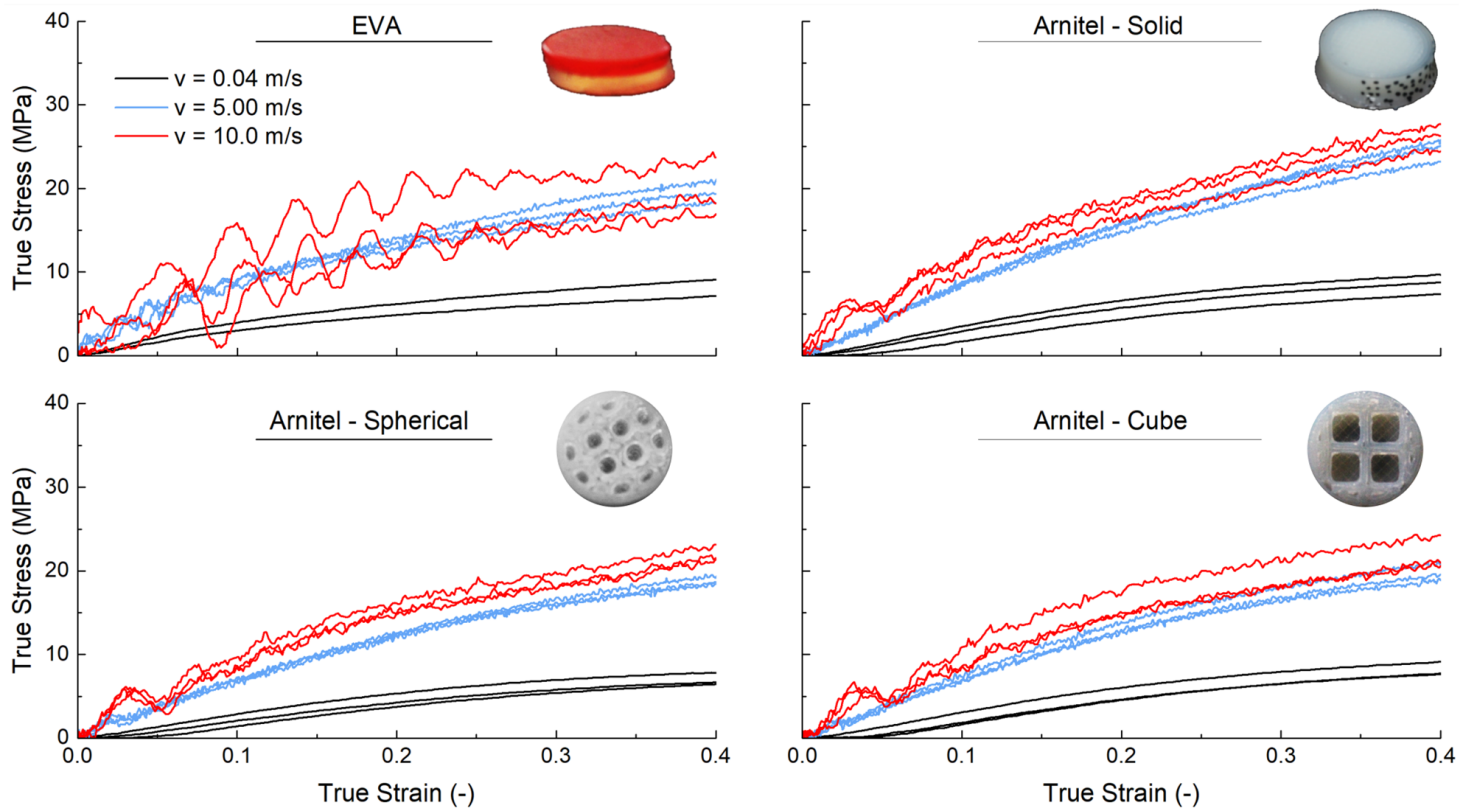
True stress-strain curves of investigated specimen configurations for different velocities.

Figure 4 shows the obtained true stress-strain results of the two different materials (EVA and Arnitel) and the different cell structures (solid, cuboid, spherical). Table 2 summarises the analysed results such as dissipated energy, specific dissipated energy and Young’s modulus for the three different loading rates.

The results suggest a strain-rate dependent behaviour of the different specimen configurations investigated. The experimental results show a standard deviation of approximately 5% independent on the loading rate. While this holds true for the 3D printed specimens, the EVA material shows a higher discrepancy of approximately 10% at the highest strain rate (10 m/s). This is also shown by the pronounced oscillation of the true stress-strain curves. The reason for the different results may be attributed to the material behaviour itself and due to the quality of the specimen manufacture.

### On the influence of strain rate on the dissipated energy

**Figure 5:**
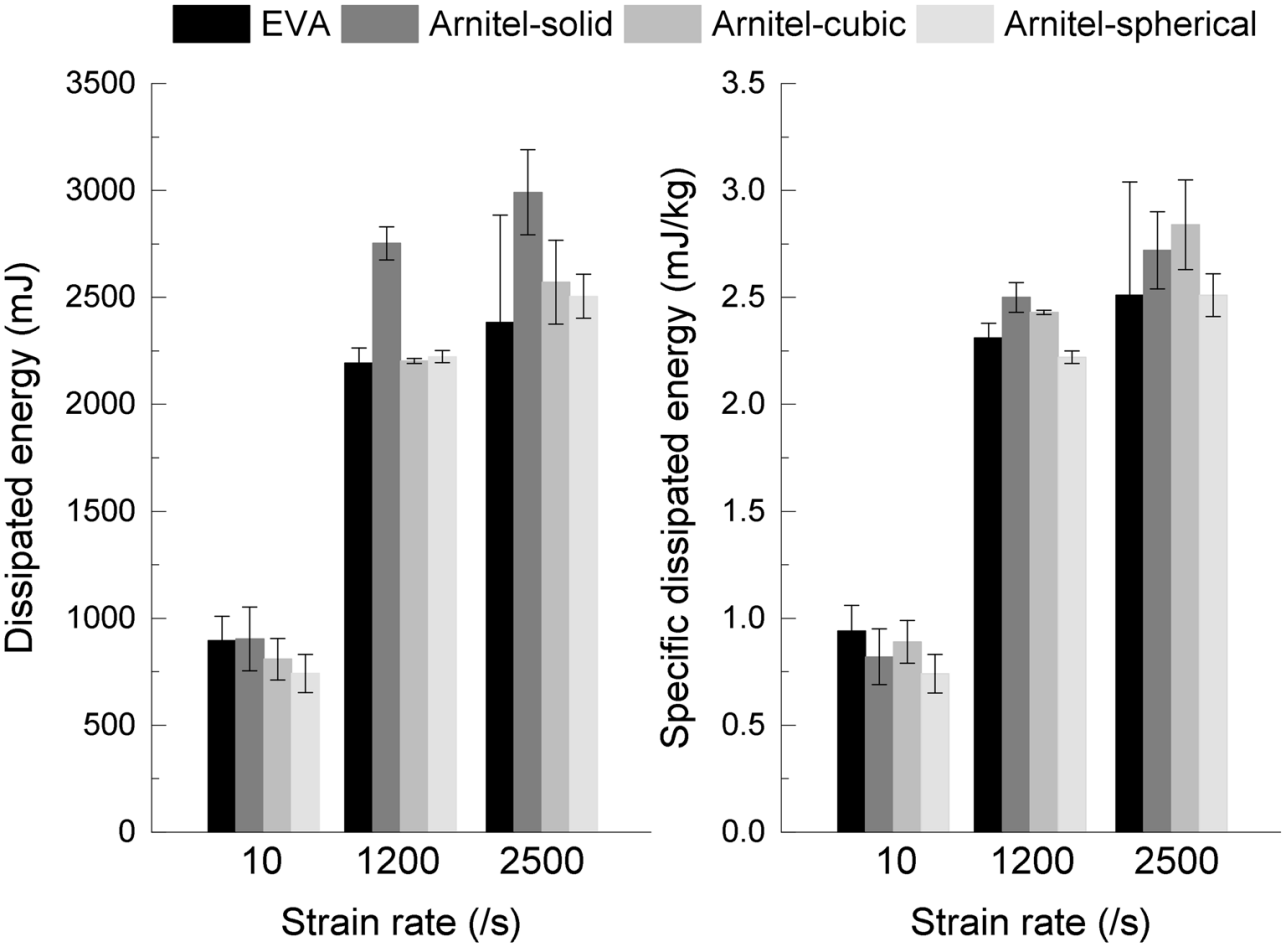
Average dissipated energy and specific dissipated energy with standard deviation by each specimen configuration at different strain rates.

The dissipated energy is calculated to understand its performance with increasing strain rate. Figure 5 together with Table 2 illustrates this. The dissipated energy shows a pronounced increase from the intermediate loading rates (0.04 m/s) to the higher rates (5 m/s). When reaching the highest investigated loading rate (10 m/s) however, a minimum increase is observed. This performance is shown independent of the different specimen configurations. This suggest, that the material is capable of dissipating energy up to a certain threshold.

**Table 2:** Impact Testing results for an applied velocity of 5 m/s taken up to 360 $\upmu$s and 10 m/s taken up to 150 $\upmu$s on the Split-Hopkinson bar and an applied velocity of 40 mm/s taken up to a strain of 20% on the Instron 8854 rig

Material	EVA	Arnitel ID 2045		
Internal structure	Solid	Solid	Spherical air cell	Cubic air cell
**Applied velocity 0.04 m/s**				
Young’s modulus (MPa) (± Stdv)	51.75 (± 0.00)	42.79 (± 3.19)	35.86 (± 1.89)	44.38 (± 0.97)
Mean difference to EVA (%)	0.00	− 17.32	− 30.70	− 14.25
Dissipated energy (J) (± Stdv)	0.89 (± 0.12)	0.90 (± 0.15)	0.74 (± 0.09)	0.81 (± 0.09)
Mean difference to EVA (%)	0.00	+ 0.88	− 17.09	− 9.65
Specific dissipated energy (mJ/kg) (± Stdv)	0.94 (± 0.12)	0.82 (± 0.13)	0.74 (± 0.09)	0.89 (± 0.10)
Mean difference to EVA (%)	0.00	− 12.88	− 21.23	− 5.26
**Applied velocity 5.00 m/s**				
Young’s Modulus (MPa) (± Stdv)	84.33 (± 5.65)	106.52 (± 3.83)	85.31 (± 0.48)	93.67 (± 2.77)
Mean difference to EVA (%)	0.00	+ 25.57	+ 1.16	+ 11.07
Dissipated energy (J) (± Stdv)	2.19 (± 0.07)	2.75 (± 0.08)	2.22 (± 0.03)	2.20 (± 0.012)
Mean difference to EVA (%)	0.00	+25.57	+1.42	+0.50
Specific dissipated energy (mJ/kg) (± Stdv)	2.31 (± 0.07)	2.50 (± 0.07)	2.22 (± 0.03)	2.43 (± 0.01)
Mean difference to EVA (%)	0.00	+ 8.45	− 3.65	+ 5.38
**Applied velocity 10.0 m/s**				
Young’s Modulus (MPa) (± Stdv)	87.85 (± 5.59)	108.01 (± 4.24)	92.91 (± 1.65)	94.62 (± 6.54)
Mean difference to EVA (%)	0.00	+ 18.40	+ 5.76	+ 7.71
Dissipated energy (J) (± Stdv)	2.38 (± 0.50)	2.99 (± 0.19)	2.51 (± 0.10)	2.57 (± 0.19)
Mean difference to EVA (%)	0.00	+ 25.46	+ 5.10	+ 7.86
Specific dissipated energy (mJ/kg) (± Stdv)	2.51 (± 0.53)	2.72 (± 0.18)	2.51 (± 0.10)	2.84 (± 0.21)
Mean difference to EVA (%)	0.00	+ 8.35	− 0.15	+ 13.10

### On the influence of internal structure on the dissipated energy

To understand the influence of the internal structure on the dissipated energy, results of the 3D printed specimens are investigated. Compared to the results of the Arnitel-solid specimens, the dissipated energy is reduced by approximately 16% independent of the cell structure (sphere and cubic air cell). This difference is also illustrated in Fig. 5 (left) where a distance between the Arnitel-solid and the two Arnitel-cells structures is observed, especially for the high-rate loading velocities.

Nevertheless, when considering the specific dissipated energy which relates the energy dissipated to the mass, a different observation is made: The cubic air cell and solid Arnitel materials show similar specific dissipated energies. The spherical air cell material, however, has a lower specific energy dissipated. This is not only shown by Table 2 but also demonstrated in Fig. 5 (right).

The potential of the cubic air cell internal structure to dissipate more energy than the solid material is in agreement with the study by Westerman et al.^19^ that found that the air cells have a beneficial effect on the energy dissipation. Despite it being easier to produce air cells via FDM than in EVA, there were still some difficulties encountered in the consistency and quality of the print. This could potentially be fixed by printing through a finer nozzle. However, this may cause other problems, relating to buckling of the filament, if it is too thin. While the cubic air cell structures increase the dissipated energy by approximately 5% when relating it to the mass, the spherical air cell structures reduces the amount by approximately 9%.

The air cells in the mouthguard might also be comparable to the air pockets created when incorporating technology into smart mouthguards. The effect of the air cells on energy dissipation could therefore provide insight into the safety of incorporating technology in a 3D printed mouthguard. As this test shows the cubic air cells are not detrimental in influencing the dissipated energy. This suggests that smart technology could be safely incorporated.

### On the influence of material on the dissipated energy

When focusing on the dissipated energy results for the different specimen configurations shown in Table 2, of the two high-rate loadings (5 m/s and 10 m/s), the Arnitel dissipated more energy than EVA. This observation is made independently of the internal structure tested. In particular, it is presented that the Arnitel-solid dissipates 25% more than the EVA specimens for the high-rate loading tests. In contrast, for the low loading rate tests the EVA specimens dissipated most energy, whilst the Arnitel specimens with the $\phi$2mm air cells dissipated the least, dissipating 17% less energy than the EVA specimens.

In general it is shown in Table 2 that all the designs tested for Arnitel dissipate more energy than the EVA specimens for the SHPB high- and medium-rate testing , ranging from 5.10% to 25.46% more. Conversely, for the testing on the Instron 8854 rig, the EVA and Arnitel-solid specimens dissipated the most energy. Nevertheless, considering the specific dissipated energy which relates the dissipated energy to the mass of the material, a different outcome is observed. In all strain rates investigated, the Arnitel-sphere perform the worst with results which are similar to EVA.

For the 10 m/s tests on the Split-Hopkinson bar, it can be seen that the Arnitel specimen with no air cells and cubic air cells were the best performing designs, dissipating 8 and 13% more energy than the EVA specimens with respect to the mass.

For the 5 m/s loading rate tests, the lowest specific energy dissipation of the Arnitel specimen configurations was the specimens with spherical air cells, and is similar to the dissipated energy of EVA specimens. This shows, that the Arnitel with spherical air cells dissipates slightly less energy when compared to the EVA material. Solid Arnitel specimens dissipate the most energy exceeding the EVA material’s ability.

In the 40 mm/s tests, the solid Arnitel specimens perform at least as well as the EVA material as the similar values of dissipated energy suggest—0.90 J and 0.89 J, respectively. However, the Arnitel specimens with spherical and cuboid air cell structures demonstrate a decrease in dissipated energy. The negative influence of the air cell structures on the dissipated energy in the low-rate regimes might be due to the quality of the print or due to the air cell geometry.

### Specimen failure

**Figure 6:**
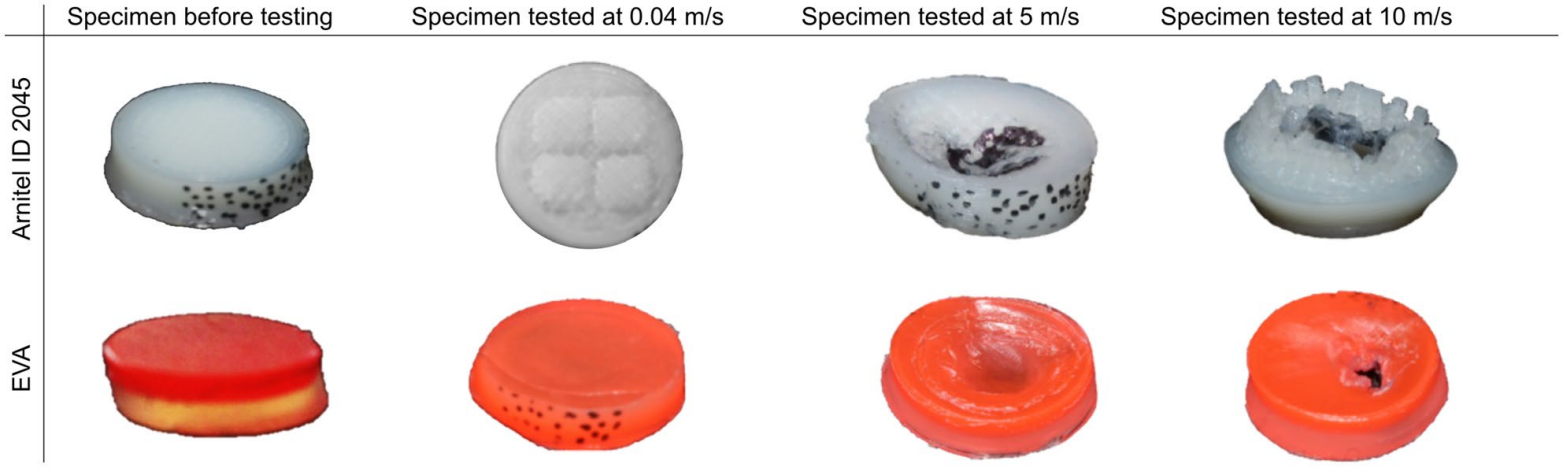
Overview of specimens before testing and after testing for three different strain rates.

Figure 6 provides an overview of the selected failed specimens for the EVA and Arnitel material. The mouthguards are not likely to experience the force required to cause such failure when used during sports. It can be seen that the EVA deforms in a very different way to Arnitel. Despite printing with 100% infill, it can be seen that Arnitel starts to fragment, breaking up into cylinders. This is consistent with the findings of Sood et al.’s study, which found that failure of a 3D printed material is likely to occur due to rupturing of rasters^33^. This is undesirable in a mouthguard as these fragments could be at risk of breaking off the mouthguard, and being ingested. Even if these fragments do not break off, they produce sharp edges, which could injure the user. Yet, the likelihood of this is low as no large fragments were seen coming off the material during testing. In contrast, despite significant deformations, the EVA specimen deforms smoothly, and does not show signs of breaking up. It may seem safer that EVA deforms more evenly. Regardless of this, it may be preferable to use a material that shows more obvious deformation. Mouthguards should not be used once the material has failed as they will not be able to dissipate sufficient energy from subsequent impacts. Therefore, the significant deformation of Arnitel may be beneficial as it makes it clearer to the user that the mouthguard has failed.

## Conclusion

In conclusion, this paper has performed experimentation to assess the possibility of 3D printing mouthguards and to investigate the effect of different internal structures. The following conclusions can be drawn:

1. Experiments for EVA and 3D printed Arnitel ID 2045 specimens at low and high strain rates reveal a clear positive strain rate dependent behaviour.
2. It was found that at medium and high strain rates, the 3D printed material performed better than the current material used in mouthguards. However, the low strain rate testing demonstrates that EVA and solid 3D printed specimens perform better when comparing to specimens with air cell structures. This suggests a strain-rate dependent failure performance of specimens with air cell structures.
3. The effect of air inclusions had varying results, depending on the strain rate of the testing. Despite this, the results indicate that 3D printing mouthguards is a feasible option and will allow some of the issues encountered with current mouthguards to be solved.
4. One limitation of this method of testing is that it is not possible to calculate the energy dissipated as a percentage of the total impact energy or the reduction in transmitted force; theses values are quoted in most other papers on the topic. However, it has provided a good comparison between a potential new mouthguard material and the material currently used. Further investigation would be required to provide these values.
